# African swine fever in the Lithuanian wild boar population in 2018: a snapshot

**DOI:** 10.1186/s12985-020-01422-x

**Published:** 2020-10-07

**Authors:** Arnoldas Pautienius, Katja Schulz, Christoph Staubach, Juozas Grigas, Ruta Zagrabskaite, Jurate Buitkuviene, Rolandas Stankevicius, Zaneta Streimikyte, Vaidas Oberauskas, Dainius Zienius, Algirdas Salomskas, Carola Sauter-Louis, Arunas Stankevicius

**Affiliations:** 1 Immunology Laboratory, Department of Anatomy and Physiology, Faculty of Veterinary Medicine, Lithuanian University of Health Sciences, Tilzes Str. 18, Kaunas, Lithuania; 2 Friedrich-Loeffler-Institut, Federal Research Institute for Animal Health, Institute of Epidemiology, Südufer 10, 17493 Greifswald-Insel Riems, Germany; 3 National Food and Veterinary Risk Assessment Institute, J. Kairiukscio Str. 10, Vilnius, Lithuania; 4 Department of Animal Breeding and Nutrition, Faculty of Animal Husbandry Technology, Lithuanian University of Health Sciences, Tilzes Str. 18, Kaunas, Lithuania; 5 Faculty of Veterinary Medicine, Institute of Microbiology and Virology, Lithuanian University of Health Sciences, Tilzes Str. 18, Kaunas, Lithuania; 6 Department of Pathobiology, Faculty of Veterinary Medicine, Lithuanian University of Health Sciences, Tilzes Str. 18, Kaunas, Lithuania

**Keywords:** Prevalence, African swine fever virus, Wild boar, Surveillance

## Abstract

The first cases of African swine fever (ASF) were detected in the Lithuanian wild boar population in 2014. Since then, the disease spread slowly through the whole country, affecting both, wild boar and domestic pigs. In the other Baltic states, which both are also affected by ASF since 2014, the recent course of ASF prevalence suggests that the countries might be well under way of disease elimination. In contrast, in Lithuania the epidemic seems to be still in full progress. In the present study, we aimed to extend a previous prevalence study in Lithuania. Looking at ASF virus (ASFV) and seroprevalence estimates of wild boar in all months of 2018 and in all affected municipalities in Lithuania, the course of ASF was evaluated on a temporal and spatial scale. A non-spatial beta-binomial model was used to correct for under- or overestimation of the average prevalence estimates. Within 2018 no big differences between the prevalence estimates were seen over time. Despite of the lower sample size, highest ASFV prevalence estimates were found in dead wild boar, suggesting higher detection rates through passive surveillance than through active surveillance. Accordingly, with the maximum prevalence of 87.5% in May 2018, the ASFV prevalence estimates were very high in wild boar found dead. The number of samples originating from hunted animals (active surveillance) predominated clearly. However, the ASFV prevalence in those animals was lower with a maximum value of 2.1%, emphasizing the high value of passive surveillance. A slight increase of the seroprevalence in hunted wild boar could be seen over time. In the center of Lithuania, a cluster of municipalities with high ASFV and seroprevalence estimates was found. The results of the study indicate that ASFV is still circulating within the Lithuanian wild boar population, constituting a permanent risk of disease transmission into domestic pig holdings. However, additional, more recent data analyses are necessary to re-evaluate the course of ASF in Lithuania and thus, to be able to make a statement about the stage of the ASF epidemic in the country. This is of huge importance for Lithuania for evaluating control measures and their efficacy, but also for neighbouring countries to assess the risk of disease spread from Lithuania.

## Introduction

African swine fever (ASF) emerged in Lithuania in 2014 [1, 2]. First ASF cases occurred in wild boar close to the border to Belarus in January 2014. Six months later, the first ASF outbreaks in domestic pigs were also reported from eastern Lithuania, suggesting disease introduction from Belarus [2]. Since then, the disease has spread through the wild boar population towards the West, affecting almost the whole country by the year 2020 [1, 3]. It is known that an infected wild boar population poses a risk for disease introduction into domestic pig farms [4–7], thus threatening the economy of the affected country [8]. Disease surveillance in wild boar is therefore of utmost importance to evaluate the course of the epidemic and thus to assess and, if necessary, to increase the effectiveness of the control measures implemented. Recent studies showed that ASF laboratory test results from wild boar samples and their different prevalence estimates (ASF virus (ASFV) and seroprevalence estimates) follow a similar epidemiological pattern over time depending on the particular stage of the epidemic [1, 9–11]. In case of a longer-lasting epidemic like in Estonia and Latvia, a decrease of ASFV prevalence estimates were observed, whereas the prevalence of wild boar being seropositive increased within both countries [9, 11, 12]. It is hypothesized that this course indicates the decline of circulating ASFV and a country obtaining such results might be on its way of disease elimination [9, 11, 12]. In a previous study, Lithuanian surveillance data from 2014 to 2017 yielded a clear increase in the ASFV prevalence estimates in wild boar found dead (from 20.1% in 2014 to 79.68% in 2017) whereas the average seroprevalence during 2014–2017 was low (0.45%) suggesting that the ASF epidemic in Lithuania was still in full progress by 2017 [1].

The present study aimed to extend this previous study and to gain further insight into the ASF epidemic in the Lithuanian wild boar population. Therefore, ASF surveillance data of 2018 were analysed to highlight recent changes in the epidemiology of ASF and to facilitate further, more comprehensive prevalence studies. In addition, the epidemiological course of ASF in Lithuania should be compared with the courses in the two other Baltic States. Differences in surveillance and control strategies should be identified and potentially adapted.

## Materials and methods

### ASF surveillance data

ASF wild boar surveillance data originated from the national surveillance program and were provided by the National Food and Veterinary Risk Assessment Institute. The data was obtained from active (hunted wild boar) and passive (wild boar found dead) surveillance and from all areas in Lithuania, which were affected by ASF in wild boar. In these areas, all hunted wild boar and each wild boar found dead were sampled, thus providing the greatest possible sample size. Sample processing is described in detail elsewhere [1]. In brief, blood samples from hunted animals were investigated for ASFV-specific antibodies by using a commercial blocking (Ingezim Compac 1.1. PPA K3, Spain) ELISA. Tissue samples originating from passive and active surveillance were examined for ASFV by PCR. Surveillance data was available for each month of 2018. The 10 counties of Lithuania are divided into 60 municipalities [3]. Analyses were done on municipality level. Aggregated surveillance data of 2018 was available for each of the 37 affected municipalities of Lithuania. The data set included information about the number of wild boars sampled (per month and per municipality), origin of sample (active/passive surveillance) and the type of laboratory investigation.

### Prevalence estimations

Similar to previous studies [9, 11, 12], for analyses, samples were grouped depending on their laboratory results.

Prevalence estimates were calculated for samples from hunted animals (active surveillance), which showed the following test results:

1. seropositive and PCR-negative (group 1)
2. seropositive and PCR-positive (group 2)
3. seronegative and PCR-positive (group 3).

In addition, prevalence estimates for wild boar, whose samples came from passive surveillance and were PCR-positive without any investigations for ASF antibodies (group 4) were determined. Thus, samples that were positive for either ASF antibodies or genome or both were considered as positive cases for their respective groups mentioned above.

Prevalence estimates within the different groups were calculated for each month and each municipality. Raw prevalence estimates for each group were calculated by dividing the number of samples showing the appropriate test result (e.g. seropositive and PCR-negative etc.) through the total number of samples that were investigated by ELISA and PCR (in case of passive surveillance, samples were solely examined by PCR) (Additional file 1: Table S1–S8) [9, 11].

Estimated raw prevalence and 95% confidence intervals for each group per municipality and per month were calculated by using the software package R (https://www.r-project.org).

### Model analysis

To avoid over- and underestimation of the true prevalence per spatial and time unit due to the heterogeneous sampling effort and population density, a non-spatial beta-binomial model was used. The methodology and software used to calculate the posterior point estimate of the true disease prevalence (corrected prevalence) and 95% confidence intervals for all groups per municipality and for each month of 2018 are described elsewhere [13].

## Results

Overall, 11,366 serum samples and 14,441 serum or tissue samples were tested for ASFV-specific antibodies and ASFV DNA, respectively. From the investigated samples, 11,366 samples originated from active and 3,352 samples from passive surveillance. Until the end of 2018, 37 Lithuanian municipalities out of 60 were affected by ASF cases in wild boar. For analyses within groups 1–3, the same number of samples was available since all samples, which were investigated for ASFV and ASFV-specific antibodies were included. Thus, in these groups, the highest number of samples was investigated in January 2018 (n = 1,614), whereas the lowest number of samples was investigated in April 2018 (n = 325) (Additional file 1: Tables S1, S2 and S3). The highest number of samples in 2018 came from the western municipality Telsiu r. sav. (n = 799) and the lowest number from the eastern municipality Svencioniu r. sav. (n = 1) (Additional file 1: Tables S5, S6 and S7).

The number of samples originating from passive surveillance was also highest in January (n = 647), whereas it showed the lowest value in December 2018 (n = 95) (Additional file 1: Table S4). In the central municipality Panevezio r. sav., the highest number of wild boar found dead was sampled within 2018 (n = 359). In the western municipality Kelmes r. sav., only one sample was collected in 2018 (Additional file 1: Table S8).

### Temporal analyses

#### Group 1

**Fig. 1:**
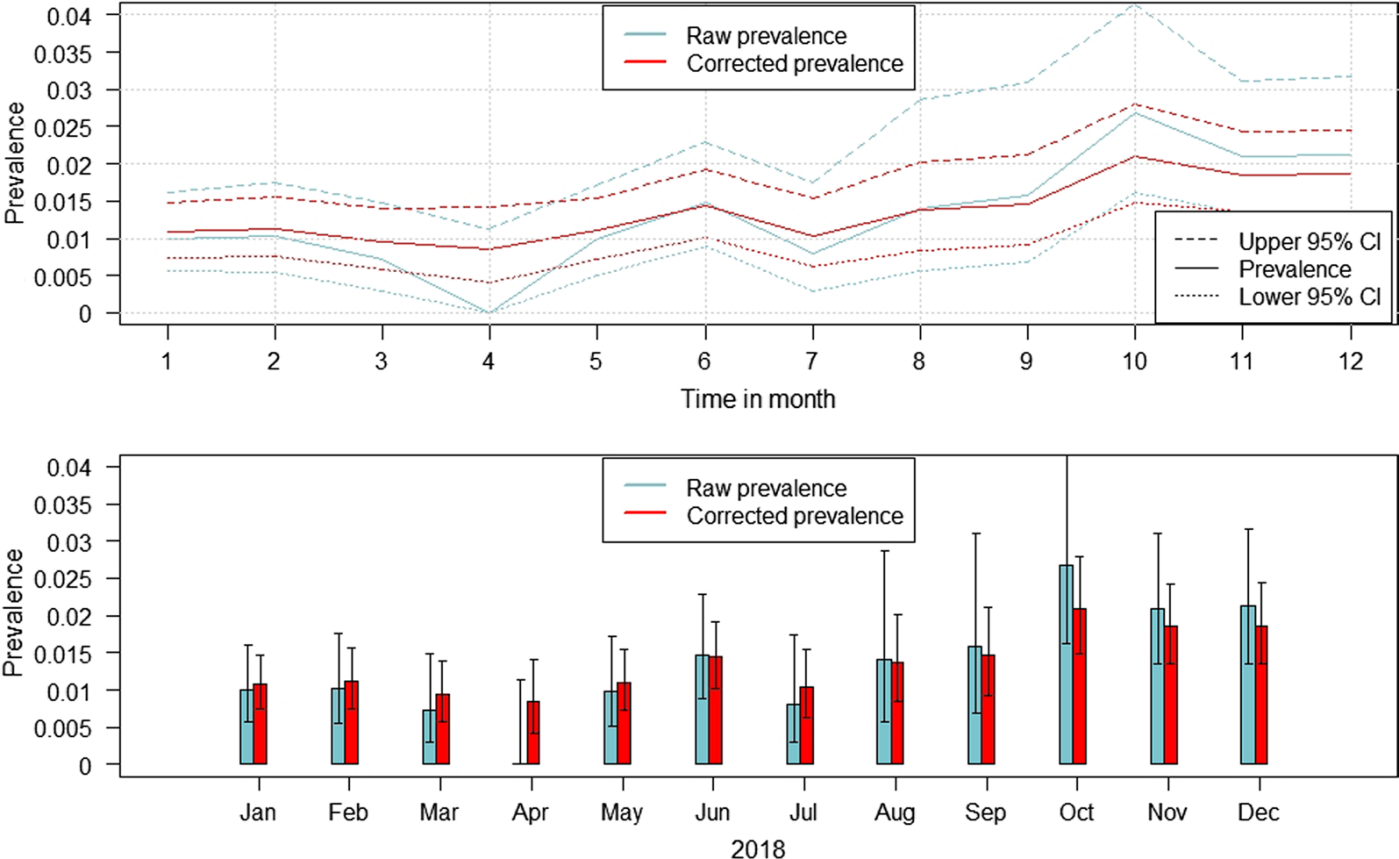
Estimated raw and corrected prevalence (calculated using a non-spatial beta-binomial model) of hunted wild boar showing a seropositive and a PCR-negative ASF sample for each month of 2018. The whiskers indicate 95% confidence intervals

In the months January–July 2018, the corrected seroprevalence was slightly higher than the raw seroprevalence. Particularly in April, the biggest difference was found (raw prevalence: 0.0%; CI 0.0–1.1% vs. corrected prevalence: 0.9%; CI 0.4–1.4%). In the remaining months of 2018, the corrected prevalence was lower than the raw prevalence estimates. A slight increase of the seroprevalence was seen from July 2018–October 2018. (Fig. 1; Additional file 1: Table S1).

#### Group 2

The prevalence of wild boar showing samples being seropositive and positive for ASFV were lower than the prevalence of the previous group with no corrected prevalence being higher than 0.4%. In most of the month, the raw and the corrected prevalence were similar or the corrected prevalence higher, respectively. However, In January, February, September and October, the corrected prevalence was lower than the raw prevalence. No clear differences were found between the months of 2018 for both, the calculated raw and corrected prevalence estimates (Additional file 1: Table S2 and Fig. S1).

#### Group 3

In group 3, prevalence estimates were calculated from wild boar yielding a seronegative and PCR-positive test result. In contrast to the two previous groups, in group 3 the highest corrected prevalence was calculated for January (2.1%; CI 1.7–2.7%), even though it was lower than the raw prevalence (2.4%; CI 1.7–3.3%). The prevalence decreased slightly until April, showed higher values in June and July but decreased again until November. In December the prevalence was similar to January (raw prevalence: 2.3%; CI 1.5–3.4% vs. corrected prevalence: 2.0%; CI 1.5–2.6%). In the 3 months with higher prevalence estimates (January, December and July), the corrected prevalence was lower than the raw prevalence whereas in the remaining month, it was slightly higher (Additional file 1: Table S3 and Fig. S2).

#### Group 4

**Fig. 2:**
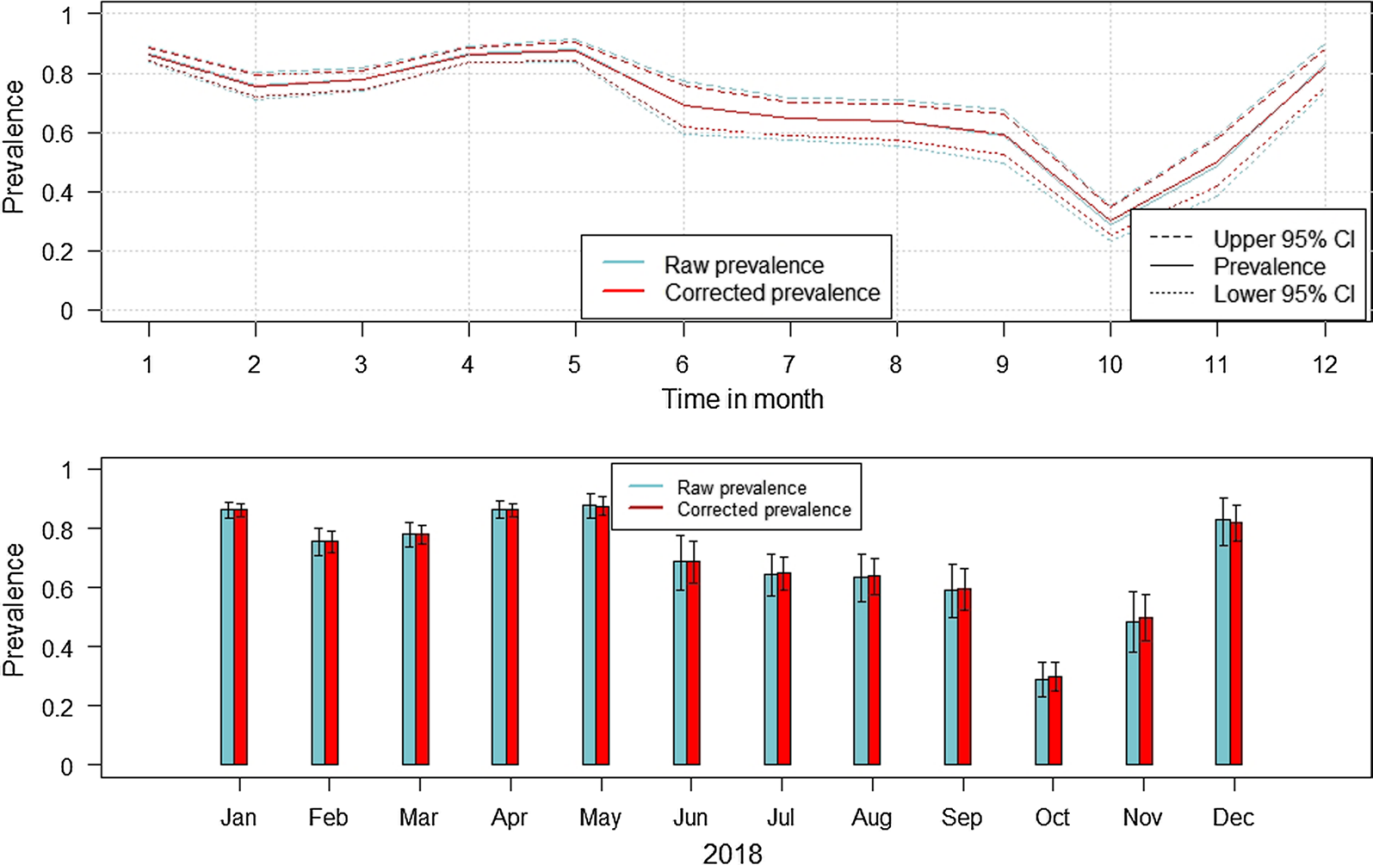
Estimated raw and corrected prevalence (calculated using a non-spatial beta-binomial model) of wild boar found dead and showing a PCR-positive ASF sample for each month of 2018. The whiskers indicate 95% confidence intervals

In group 4, only samples from passive surveillance were included. Thus, prevalence estimates for wild boar yielding a PCR-positive test result and for which no test for ASFV-specific antibodies was done, were calculated. The highest corrected prevalence estimates were found in January (86.2%; CI 83.9–88.4%), in April (86.2%; CI 83.8–88.5%) and in May (87.5%; CI 84.2–90.5%). The corrected prevalence estimates in July, August, September and October were clearly lower with the lowest prevalence in October (29.9%; CI 25.2–34.7%) (Fig. 2, Additional file 1: Table S4).

### Spatial analyses

#### Group 1

**Fig. 3:**
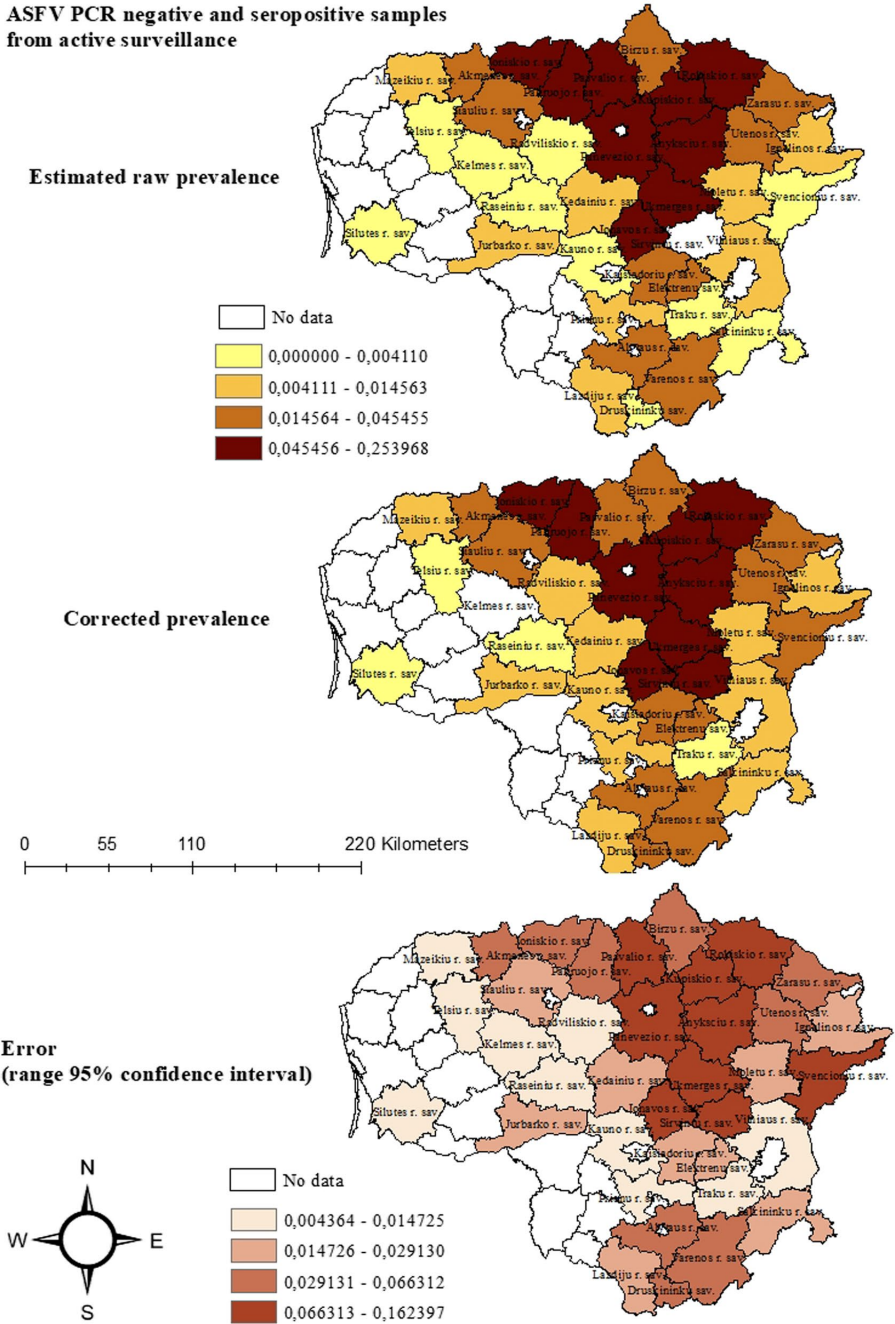
Estimated raw and corrected prevalence with 95% confidence intervals (calculated using a non-spatial beta-binomial model) of hunted wild boar showing a seropositive ASF sample, but a PCR-negative test result for each ASF-affected municipality of Lithuania

For wild boar yielding seropositive and ASFV-negative samples, in most municipalities, the raw estimated and the corrected seroprevalences were similar. Four municipalities in the northern center of Lithuania showed the highest corrected prevalences (Ukmerges r. sav. (13.8%; CI 7.1–27.7%), Panevezio r. sav. (13.2%; CI 6.3–30.5%), Sirvintu r. sav. (21.6%; CI 14.4–32.1%), Anyksciu r. sav. (20.6%; CI 13.0–33.9%). However, in all these municipalities, the sample size was relatively low, which yielded a wide range of the 95% confidence interval. Several municipalities in the western part of the country showed low prevalences but at the same time high sample sizes and therefore narrow ranges of the 95% confidence intervals (Fig. 3, Additional file 1: Table S5).

#### Group 2

The prevalence of animals being positive for both ASFV and ASF-specific antibodies was also clearly lower than in the remaining groups. The highest value was found in Panevezio r. sav., however, due to the very small sample size, the confidence interval was very wide (raw prevalence: 8.3%; CI 1.8–22.5% vs. corrected prevalence: 3.5%; CI 1.1–6.9%). In 14 municipalities, the raw prevalence was 0%; however, the corrected prevalence yielded slightly higher values. In contrast, in the six municipalities with the highest raw prevalences (Kupiskio r. sav., Anyksciu r. sav., Druskininku sav., Pakruojo r. sav., Ukmerges r. sav., Panevezio r. sav.), the corrected prevalences were slightly lower (Additional file 1: Table S6 and Fig. S3).

#### Group 3

The highest corrected prevalence for hunted wild boar showing a seronegative but PCR-positive test result was found in the municipality Pakruojo r. sav. in the center (5.3%; CI 3.1–8.0%). In contrast to the prevalences of the other groups, the corrected prevalence was also higher in Zarasu r. sav. in the east of the country (3.1%; CI 1.5–5.3%) and in Kedainiu r. sav. in the center (3.4%; CI 2.2–4.7%). The raw and the corrected prevalences were very similar and in cases of a high raw prevalence, the corrected prevalence was slightly lower, whereas in cases of a low raw prevalence, the corrected prevalence was slightly higher (Additional file 1: Table S7 and Fig. S4).

#### Group 4

Similar to the temporal analysis of this group, also on spatial level, the raw and the corrected prevalences were very high with small confidence intervals. The highest corrected prevalence was found in Panevezio r. sav. (99.1%; CI 98.1–99.7%). The lowest corrected prevalence was found in Svencioniu r. sav. in the east but still with a value of 21.9% (CI 17–27.2%). In the southern municipality Druskininku sav., the raw prevalence was 100% (CI 63.1–100%), however, due to the small sample size, the corrected prevalence was 89% (CI 72.7–98.6%) (Additional file 1: Table S8 and Fig. S5).

## Discussion

Lithuania is affected by ASF since 2014 [2]. Several studies suggest that the course of disease within wild boar populations follows similar patterns in different countries [9, 11]. It was found that after a period of approximately 100 days, in which infected animals can be tested positive for ASFV and ASFV-specific antibodies at the same time, surviving animals are seropositive for an unknown period of time [14, 15]. Due to a long half-life of ASFV-specific antibodies, it might well be that this period lasts for several years [16, 17]. In countries in which an increase of seropositive and simultaneously a decrease in ASFV-positive wild boar was found, a decline of ASF incidence and a subsiding stage of the epidemic was hypothesized [9, 11]. Following these observations and as a logical extension of a previous Lithuanian study [1], we aimed to analyse ASF surveillance data in wild boar and thereby to evaluate the course of the disease in 2018.

Taking the assumed role of the individual test results within the course of ASF into account, we divided the samples in different groups depending on the results. To see the difference in the prevalence estimates between hunted animals and those who were found dead, samples were additionally divided depending on their origin (active or passive surveillance). The model was used to take the different conditions in the individual months and municipalities into account. Usually, the main hunting activities take place in the winter months. Thus, for the groups, analysing samples from active surveillance, the corrected prevalence was higher than the raw prevalence in months, in which the hunting effort is usually low (e.g. in April). Therefore, although the calculated prevalence estimates were very low in these months, the prevalence estimates obtained through the model helped to correct for such external conditions and falsified conclusions could be avoided. The same applied for the spatial analyses, where the model took the prevalence estimates of the individual municipalities in the area into account by shrinking the estimates to the global mean, therefore corrected for under- or overestimations due to different sampling behaviour or different population densities.

Between 2016 and 2017, the seroprevalence in Lithuanian wild boar increased clearly [1]. This increase seemed to continue in 2018. However, although a slight increase of the seroprevalence was seen by the end of 2018, these changes were not significant. In contrast, the difference in the mean prevalence of ASFV-positive wild boar between 2017 and 2018 was negligible [1]. Also within 2018, despite of the seasonal fluctuations, no significant change was seen in the ASFV prevalence, particularly not in wild boar samples from hunted wild boar. The higher ASFV prevalence estimates in the summer months corresponds to the seasonal patterns of ASFV, which were found in other countries [12, 18]. Due to a relatively high sample size and a generally low difference within the individual months, the raw and the corrected prevalence estimates were very similar in wild boar samples originating from passive surveillance. These results support results from a previous study with Lithuanian wild boar data [3]. However, in October and November, the prevalence estimates were lower than in the other months. A similar course was seen in Estonia [9]. These results might be due to a lower number of young wild boar being present in these months, which results from the natural reproductive cycle of wild boar. It is assumed that in the field, the case-fatality ratio is higher in wild boar younger than 1 year [10, 19]. However, due to the lack of information on age of the sampled animals in the present data set, no statements were possible regarding the age distribution within the different results.

Both, the raw and the corrected prevalence estimates of wild boar found dead and being PCR-positive for ASFV were much higher than the prevalence estimates of ASFV positive hunted wild boar. Due to the relative high sample size (mean = 279) and the high number of positive samples, the confidence intervals were smaller and so were the differences between the raw and corrected prevalence within the single months (Additional file 1: Table S4). These findings show the higher ASF detection rate in animals found dead and correlate with results from Estonia and Latvia [7, 10, 11, 19, 20]. In addition, they emphasise once more the need to focus on passive surveillance [3, 9, 10, 19, 21, 22]. The high ASFV prevalence estimates in wild boar found dead and the consistent ASFV prevalence estimates over the year suggest that ASFV is still circulating within the Lithuanian wild boar population. By the end of 2018, the prevalence estimates from wild boar showing ASFV- and seropositive test results at the same time hardly differ from the prevalence estimates at the beginning of 2018. These result support the hypothesis that circulating ASFV is still considerably present, as animals showing such a result are very likely to have become infected within the last 3 months [9, 14, 15]. The spatial analyses showed similar patterns. In the central municipalities, ASFV and seroprevalence where high. However, the 95% confidence interval in these areas was very wide, indicating a small sample size and thus a considerable uncertainty regarding the true prevalence. However, all of these municipalities have borders with previously, highly affected areas [1]. Therefore, also the corrected prevalence estimates were clearly higher in the central municipalities than in the neighbouring municipalities. In a previous study, similar results were obtained and high prevalence estimates were mainly found in Anykščiai et al. [3]. A higher wild boar density in these areas is described as a potential cause for the geographical distribution of prevalence estimates [3]. However, in our study, population density was not included in the analyses as well as other risk-factors like different sampling strategies, human activities and other potential factors supporting the spread of ASF. To pinpoint the true reasons for this ASF cluster in the center of Lithuania, further, more comprehensive studies are necessary.

## Conclusion

To evaluate the course of ASF and the effectiveness of disease control in Lithuania, a prevalence study regarding ASF in the Lithuanian wild boar population in 2018 was performed. The results of the present study based on 2018 data show that in contrast to similar studies from other countries in the region, ASF was still active in Lithuania in 2018. Therefore, it is of utmost importance to maintain or even to adapt intensive surveillance and control measures and to regularly evaluate the course of ASF on the basis of laboratory data. Further prevalence studies are necessary to assess the current situation of ASF in Lithuania, not only for Lithuania authorities for evaluating the success of control measures but also for neighbouring countries to estimate the risk of disease introduction from Lithuania.

## Supplementary information

**Additional file1. Table S1**: Number of wild boar samples, which were investigated by ELISA to detected ASF specific antibodies and by PCR to detect ASF virus genome. Number of samples from active surveillance, which resulted in a seropositive test results for ASF and a negative PCR test result and the estimated raw and corrected prevalence (calculated using a non-spatial beta-binomial model) including the 95% confidence intervals for each month of 2018. **Table S2**: Number of wild boar samples, which were investigated by ELISA to detected ASF specific antibodies and by PCR to detect ASF virus genome. Number of samples from active surveillance, which resulted in a seropositive test results for ASF and a positive PCR test result and the estimated raw and corrected prevalence (calculated using a non-spatial beta-binomial model) including the 95% confidence intervals for each month of 2018. **Table S3**: Number of wild boar samples, which were investigated by ELISA to detected ASF specific antibodies and by PCR to detect ASF virus genome. Number of samples from active surveillance, which resulted in a positive PCR but a negative ELISA test result and the estimated raw and corrected prevalence (calculated using a non-spatial beta-binomial model) including the 95% confidence intervals for each month of 2018. **Table S4**: Number of wild boar samples, which were investigated only by PCR to detect ASF virus genome. Number of samples from passive surveillance, which resulted in a positive PCR test result and the estimated raw and corrected prevalence (calculated using a non-spatial beta-binomial model) including the 95% confidence intervals for each month of 2018. **Table S5**: Number of wild boar samples, which were investigated by ELISA to detected ASF specific antibodies and by PCR to detect ASF virus genome. Number of samples from active surveillance, which resulted in a seropositive test results for ASF and a negative PCR test result and the estimated raw and corrected prevalence (calculated using a non-spatial beta-binomial model) including the 95% confidence intervals for each ASF-affected municipality of Lithuania. **Table S6**: Number of wild boar samples, which were investigated by ELISA to detected ASF specific antibodies and by PCR to detect ASF virus genome. Number of samples from active surveillance, which resulted in a seropositive test results for ASF and a positive PCR test result and the estimated raw and corrected prevalence (calculated using a non-spatial beta-binomial model) including the 95% confidence intervals for each ASF-affected municipality of Lithuania. **Table S7**: Number of wild boar samples, which were investigated by ELISA to detected ASF specific antibodies and by PCR to detect ASF virus genome. Number of samples from active surveillance, which resulted in a positive PCR but a negative ELISA test result and the estimated raw and corrected prevalence (calculated using a non-spatial beta-binomial model) including the 95% confidence intervals for each ASF-affected municipality of Lithuania. **Table S8**: Number of wild boar samples, which were investigated only by PCR to detect ASF virus genome. Number of samples from passive surveillance, which resulted in a positive PCR test result and the estimated raw and corrected prevalence (calculated using a non-spatial beta-binomial model) including the 95% confidence intervals for each ASF-affected municipality of Lithuania. **Figure S1**: Estimated raw and corrected prevalence (calculated using a non-spatial beta-binomial model) of hunted wild boar showing an ELISA and a PCR-positive test result for each month of 2018. The whiskers indicate 95% confidence intervals. **Figure S2**: Estimated raw and corrected prevalence (calculated using a non-spatial beta-binomial model) of hunted wild boar showing an ELISA-negative but a PCR-positive test result for each month of 2018. The whiskers indicate 95% confidence intervals. **Figure S3**: Estimated raw and corrected prevalence with 95% confidence intervals (calculated using a non-spatial beta-binomial model) of hunted wild boar showing a sero- and PCR-positive ASF sample result for each ASF-affected municipality of Lithuania. **Figure S4**: Estimated raw and corrected prevalence with 95% confidence intervals (calculated using a non-spatial beta-binomial model) of hunted wild boar showing a PCR-positive and a seronegative ASF sample result for each ASF-affected municipality of Lithuania. **Figure S5**: Estimated raw and corrected prevalence with 95% confidence intervals (calculated using a non-spatial beta-binomial model) of wild boar found dead showing a PCR-positive ASF sample result for each ASF-affected municipality of Lithuania.

## Data Availability

The data used are available in the Additional file 1.

## Abbreviations

ASF: African swine fever
ASFV: African swine fever virus
