# Supplementary material for: African swine fever in the Lithuanian wild boar population in 2018: a snapshot
Source: Virol J. 2020 Oct 7;17:148. doi: 10.1186/s12985-020-01422-x (PMC7542886; doi:10.1186/s12985-020-01422-x)
Supplement: Supplementary file 1 — Additional file1. Table S1: Number of wild boar samples, which were investigated by ELISA to detected ASF specific antibodies and by PCR to detect ASF virus genome. Number of samples from active surveillance, which resulted in a seropositive test results for ASF and a negative PCR test result and the estimated raw and corrected prevalence (calculated using a non-spatial beta-binomial model) including the 95% confidence intervals for each month of 2018. Table S2: Number of wild boar samples, which were investigated by ELISA to detected ASF specific antibodies and by PCR to detect ASF virus genome. Number of samples from active surveillance, which resulted in a seropositive test results for ASF and a positive PCR test result and the estimated raw and corrected prevalence (calculated using a non-spatial beta-binomial model) including the 95% confidence intervals for each month of 2018. Table S3: Number of wild boar samples, which were investigated by ELISA to detected ASF specific antibodies and by PCR to detect ASF virus genome. Number of samples from active surveillance, which resulted in a positive PCR but a negative ELISA test result and the estimated raw and corrected prevalence (calculated using a non-spatial beta-binomial model) including the 95% confidence intervals for each month of 2018. Table S4: Number of wild boar samples, which were investigated only by PCR to detect ASF virus genome. Number of samples from passive surveillance, which resulted in a positive PCR test result and the estimated raw and corrected prevalence (calculated using a non-spatial beta-binomial model) including the 95% confidence intervals for each month of 2018. Table S5: Number of wild boar samples, which were investigated by ELISA to detected ASF specific antibodies and by PCR to detect ASF virus genome. Number of samples from active surveillance, which resulted in a seropositive test results for ASF and a negative PCR test result and the estimated raw and corrected prevalence (calc [file 12985_2020_1422_MOESM1_ESM.docx]

**Supplementary material**

**Table S1:** Number of wild boar samples, which were investigated by ELISA to detected ASF specific antibodies and by PCR to detect ASF virus genome. Number of samples from active surveillance, which resulted in a seropositive test results for ASF and a negative PCR test result and the estimated raw and corrected prevalence (calculated using a non-spatial beta-binomial model) including the 95% confidence intervals for each month of 2018.

| Months | Number of wild boar showing a seropositive and a PCR-negative ASF sample | Number of samples | Raw prevalence | Lower 95% Confidence interval | Upper 95% Confidence interval | Corrected prevalence | Lower 95% Confidence interval | Upper 95% Confidence interval |
| --- | --- | --- | --- | --- | --- | --- | --- | --- |
| 1 | 16 | 1.614 | 1.0% | 0.6% | 1.6% | 1.1% | 0.8% | 1.5% |
| 2 | 13 | 1.265 | 1.0% | 0.6% | 1.8% | 1.1% | 0.8% | 1.6% |
| 3 | 7 | 971 | 0.7% | 0.3% | 1.5% | 1.0% | 0.6% | 1.4% |
| 4 | 0 | 325 | 0.0% | 0.0% | 1.1% | 0.9% | 0.4% | 1.4% |
| 5 | 12 | 1.216 | 1.0% | 0.5% | 1.7% | 1.1% | 0.7% | 1.5% |
| 6 | 19 | 1.288 | 1.5% | 0.9% | 2.3% | 1.4% | 1.0% | 1.9% |
| 7 | 6 | 745 | 0.8% | 0.3% | 1.7% | 1.0% | 0.6% | 1.5% |
| 8 | 7 | 500 | 1.4% | 0.6% | 2.9% | 1.4% | 0.8% | 2.0% |
| 9 | 8 | 505 | 1.6% | 0.7% | 3.1% | 1.5% | 0.9% | 2.1% |
| 10 | 19 | 710 | 2.7% | 1.6% | 4.2% | 2.1% | 1.5% | 2.8% |
| 11 | 24 | 1.144 | 2.1% | 1.4% | 3.1% | 1.9% | 1.4% | 2.4% |
| 12 | 23 | 1.083 | 2.1% | 1.4% | 3.2% | 1.9% | 1.4% | 2.4% |

**Table S2:** Number of wild boar samples, which were investigated by ELISA to detected ASF specific antibodies and by PCR to detect ASF virus genome. Number of samples from active surveillance, which resulted in a seropositive test results for ASF and a positive PCR test result and the estimated raw and corrected prevalence (calculated using a non-spatial beta-binomial model) including the 95% confidence intervals for each month of 2018.

| Months | Number of wild boar showing a seropositive and a PCR-positive ASF sample | Number of samples | Raw prevalence | Lower 95% Confidence interval | Upper 95% Confidence interval | Corrected prevalence | Lower 95% Confidence interval | Upper 95% Confidence interval |
| --- | --- | --- | --- | --- | --- | --- | --- | --- |
| 1 | 10 | 1.614 | 0.6% | 0.3% | 1.1% | 0.4% | 0.3% | 0.6% |
| 2 | 5 | 1.265 | 0.4% | 0.1% | 0.9% | 0.4% | 0.2% | 0.5% |
| 3 | 3 | 971 | 0.3% | 0.1% | 0.9% | 0.3% | 0.2% | 0.5% |
| 4 | 1 | 325 | 0.3% | 0.0% | 1.7% | 0.3% | 0.2% | 0.5% |
| 5 | 2 | 1.216 | 0.2% | 0.0% | 0.6% | 0.3% | 0.2% | 0.4% |
| 6 | 1 | 1.288 | 0.1% | 0.0% | 0.4% | 0.2% | 0.1% | 0.4% |
| 7 | 1 | 745 | 0.1% | 0.0% | 0.8% | 0.3% | 0.2% | 0.5% |
| 8 | 1 | 500 | 0.2% | 0.0% | 1.1% | 0.3% | 0.2% | 0.5% |
| 9 | 2 | 505 | 0.4% | 0.1% | 1.4% | 0.3% | 0.2% | 0.5% |
| 10 | 6 | 710 | 0.9% | 0.3% | 1.8% | 0.4% | 0.3% | 0.6% |
| 11 | 3 | 1.144 | 0.3% | 0.1% | 0.8% | 0.3% | 0.2% | 0.5% |
| 12 | 2 | 1.083 | 0.2% | 0.0% | 0.7% | 0.3% | 0.2% | 0.4% |

**Table S3:** Number of wild boar samples, which were investigated by ELISA to detected ASF specific antibodies and by PCR to detect ASF virus genome. Number of samples from active surveillance, which resulted in a positive PCR but a negative ELISA test result and the estimated raw and corrected prevalence (calculated using a non-spatial beta-binomial model) including the 95% confidence intervals for each month of 2018.

| Months | Number of wild boar showing a PCR-positive and a seronegative ASF sample | Number of samples | Raw prevalence | Lower 95% Confidence interval | Upper 95% Confidence interval | Corrected prevalence | Lower 95% Confidence interval | Upper 95% Confidence interval |
| --- | --- | --- | --- | --- | --- | --- | --- | --- |
| 1 | 39 | 1.614 | 2.4% | 1.7% | 3.3% | 2.2% | 1.7% | 2.7% |
| 2 | 14 | 1.265 | 1.1% | 0.6% | 1.9% | 1.2% | 0.8% | 1.6% |
| 3 | 5 | 971 | 0.5% | 0.2% | 1.2% | 0.8% | 0.4% | 1.2% |
| 4 | 1 | 325 | 0.3% | 0.0% | 1.7% | 0.9% | 0.4% | 1.5% |
| 5 | 9 | 1.216 | 0.7% | 0.3% | 1.4% | 0.9% | 0.6% | 1.3% |
| 6 | 18 | 1.288 | 1.4% | 0.8% | 2.2% | 1.4% | 1.0% | 1.9% |
| 7 | 15 | 745 | 2.0% | 1.1% | 3.3% | 1.7% | 1.2% | 2.4% |
| 8 | 5 | 500 | 1.0% | 0.3% | 2.3% | 1.2% | 0.7% | 1.8% |
| 9 | 5 | 505 | 1.0% | 0.3% | 2.3% | 1.1% | 0.7% | 1.8% |
| 10 | 8 | 710 | 1.1% | 0.5% | 2.2% | 1.2% | 0.7% | 1.8% |
| 11 | 12 | 1.144 | 1.1% | 0.5% | 1.8% | 1.1% | 0.7% | 1.6% |
| 12 | 25 | 1.083 | 2.3% | 1.5% | 3.4% | 2.0% | 1.5% | 2.6% |

**Table S4:** Number of wild boar samples, which were investigated only by PCR to detect ASF virus genome. Number of samples from passive surveillance, which resulted in a positive PCR test result and the estimated raw and corrected prevalence (calculated using a non-spatial beta-binomial model) including the 95% confidence intervals for each month of 2018.

| Months | Number of wild boar showing a PCR-positive ASF sample | Number of samples | Raw prevalence | Lower 95% Confidence interval | Upper 95% Confidence interval | Corrected prevalence | Lower 95% Confidence interval | Upper 95% Confidence interval |
| --- | --- | --- | --- | --- | --- | --- | --- | --- |
| 1 | 559 | 647 | 86.4% | 83.5% | 89.0% | 86.2% | 83.9% | 88.4% |
| 2 | 274 | 362 | 75.7% | 70.9% | 80.0% | 75.6% | 71.8% | 79.2% |
| 3 | 351 | 450 | 78.0% | 73.9% | 81.7% | 77.9% | 74.6% | 81.0% |
| 4 | 516 | 597 | 86.4% | 83.4% | 89.1% | 86.2% | 83.8% | 88.5% |
| 5 | 249 | 283 | 88.0% | 83.6% | 91.5% | 87.5% | 84.2% | 90.5% |
| 6 | 73 | 106 | 68.9% | 59.1% | 77.5% | 68.9% | 61.6% | 75.8% |
| 7 | 122 | 189 | 64.6% | 57.3% | 71.4% | 64.7% | 59.0% | 70.2% |
| 8 | 99 | 156 | 63.5% | 55.4% | 71.0% | 63.7% | 57.5% | 69.8% |
| 9 | 73 | 124 | 58.9% | 49.7% | 67.6% | 59.4% | 52.3% | 66.4% |
| 10 | 70 | 244 | 28.7% | 23.1% | 34.8% | 29.9% | 25.2% | 34.7% |
| 11 | 48 | 99 | 48.5% | 38.3% | 58.8% | 49.9% | 42.0% | 57.9% |
| 12 | 79 | 95 | 83.2% | 74.1% | 90.1% | 82.1% | 75.6% | 87.9% |

**Table S5:** Number of wild boar samples, which were investigated by ELISA to detected ASF specific antibodies and by PCR to detect ASF virus genome. Number of samples from active surveillance, which resulted in a seropositive test results for ASF and a negative PCR test result and the estimated raw and corrected prevalence (calculated using a non-spatial beta-binomial model) including the 95% confidence intervals for each ASF-affected municipality of Lithuania.

| Area | Number of wild boar showing a seropositive and a PCR-negative ASF sample | Number of samples | Raw prevalence | Lower 95% Confidence interval | Upper 95% Confidence interval | Corrected prevalence | Lower 95% Confidence interval | Upper 95% Confidence interval |
| --- | --- | --- | --- | --- | --- | --- | --- | --- |
| Sirvintu r. sav. | 16 | 63 | 25.4% | 15.3% | 37.9% | 21.6% | 14.4% | 32.1% |
| Anyksciu r. sav. | 13 | 52 | 25.0% | 14.0% | 39.0% | 20.6% | 13.0% | 33.9% |
| Ukmerges r. sav. | 7 | 41 | 17.1% | 7.2% | 32.1% | 13.8% | 7.1% | 27.7% |
| Panevezio r. sav. | 6 | 36 | 16.7% | 6.4% | 32.8% | 13.2% | 6.3% | 30.5% |
| Rokiskio r. sav. | 6 | 42 | 14.3% | 5.4% | 28.5% | 11.8% | 5.6% | 20.5% |
| Jonavos r. sav. | 5 | 40 | 12.5% | 4.2% | 26.8% | 10.4% | 4.5% | 18.8% |
| Kupiskio r. sav. | 5 | 51 | 9.8% | 3.3% | 21.4% | 8.6% | 3.7% | 17.6% |
| Pakruojo r. sav. | 7 | 131 | 5.3% | 2.2% | 10.7% | 5.2% | 2.6% | 13.9% |
| Joniskio r. sav. | 7 | 146 | 4.8% | 2.0% | 9.6% | 4.8% | 2.3% | 7.9% |
| Pasvalio r. sav. | 2 | 44 | 4.6% | 0.6% | 15.5% | 4.5% | 1.1% | 10.1% |
| Svencioniu r. sav. | 0 | 1 | 0.0% | 0.0% | 97.5% | 4.0% | 0.0% | 17.1% |
| Birzu r. sav. | 2 | 61 | 3.3% | 0.4% | 11.4% | 3.5% | 0.8% | 9.7% |
| Kaisiadoriu r. sav. | 12 | 409 | 2.9% | 1.5% | 5.1% | 3.0% | 1.8% | 4.8% |
| Alytaus r. sav. | 5 | 175 | 2.9% | 0.9% | 6.5% | 3.0% | 1.3% | 6.0% |
| Siauliu r. sav. | 10 | 344 | 2.9% | 1.4% | 5.3% | 3.0% | 1.7% | 6.0% |
| Akmenes r. sav. | 4 | 153 | 2.6% | 0.7% | 6.6% | 2.8% | 1.0% | 6.8% |
| Utenos r. sav. | 6 | 254 | 2.4% | 0.9% | 5.1% | 2.5% | 1.1% | 5.2% |
| Varenos r. sav. | 3 | 133 | 2.3% | 0.5% | 6.5% | 2.4% | 0.8% | 5.9% |
| Zarasu r. sav. | 2 | 128 | 1.6% | 0.2% | 5.5% | 1.8% | 0.4% | 5.1% |
| Druskininku sav. | 0 | 22 | 0.0% | 0.0% | 15.4% | 1.6% | 0.0% | 12.0% |
| Elektrenu sav. | 3 | 206 | 1.5% | 0.3% | 4.2% | 1.6% | 0.5% | 3.9% |
| Lazdiju r. sav. | 2 | 166 | 1.2% | 0.2% | 4.3% | 1.4% | 0.3% | 3.2% |
| Kedainiu r. sav. | 5 | 441 | 1.1% | 0.4% | 2.6% | 1.2% | 0.5% | 2.5% |
| Ignalinos r. sav. | 1 | 120 | 0.8% | 0.0% | 4.6% | 1.2% | 0.2% | 3.1% |
| Jurbarko r. sav. | 3 | 302 | 1.0% | 0.2% | 2.9% | 1.1% | 0.4% | 2.7% |
| Moletu r. sav. | 3 | 336 | 0.9% | 0.2% | 2.6% | 1.0% | 0.3% | 2.5% |
| Vilniaus r. sav. | 4 | 450 | 0.9% | 0.2% | 2.3% | 1.0% | 0.4% | 1.9% |
| Mazeikiu r. sav. | 3 | 460 | 0.7% | 0.1% | 1.9% | 0.8% | 0.2% | 1.8% |
| Prienu r. sav. | 3 | 572 | 0.5% | 0.1% | 1.5% | 0.6% | 0.2% | 1.2% |
| Salcininku r. sav. | 0 | 85 | 0.0% | 0.0% | 4.3% | 0.6% | 0.0% | 2.2% |
| Radviliskio r. sav. | 2 | 503 | 0.4% | 0.1% | 1.4% | 0.5% | 0.1% | 1.4% |
| Kauno r. sav. | 3 | 730 | 0.4% | 0.1% | 1.2% | 0.5% | 0.2% | 1.0% |
| Silutes r. sav. | 0 | 135 | 0.0% | 0.0% | 2.7% | 0.4% | 0.0% | 1.5% |
| Traku r. sav. | 1 | 412 | 0.2% | 0.0% | 1.3% | 0.4% | 0.1% | 1.3% |
| Raseiniu r. sav. | 1 | 431 | 0.2% | 0.0% | 1.3% | 0.4% | 0.1% | 0.9% |
| Telsiu r. sav. | 2 | 799 | 0.3% | 0.0% | 0.9% | 0.3% | 0.1% | 0.9% |
| Kelmes r. sav. | 0 | 472 | 0.0% | 0.0% | 0.8% | 0.1% | 0.0% | 0.5% |

**Table S6:** Number of wild boar samples, which were investigated by ELISA to detected ASF specific antibodies and by PCR to detect ASF virus genome. Number of samples from active surveillance, which resulted in a seropositive test results for ASF and a positive PCR test result and the estimated raw and corrected prevalence (calculated using a non-spatial beta-binomial model) including the 95% confidence intervals for each ASF-affected municipality of Lithuania.

| Area | Number of wild boar showing a seropositive and a PCR-positive ASF sample | Number of samples | Raw prevalence | Lower 95% Confidence interval | Upper 95% Confidence interval | Corrected prevalence | Lower 95% Confidence interval | Upper 95% Confidence interval |
| --- | --- | --- | --- | --- | --- | --- | --- | --- |
| Panevezio r. sav. | 3 | 36 | 8.3% | 1.8% | 22.5% | 3.5% | 1.1% | 6.9% |
| Pakruojo r. sav. | 6 | 131 | 4.6% | 1.7% | 9.7% | 3.3% | 1.5% | 5.7% |
| Ukmerges r. sav. | 2 | 41 | 4.9% | 0.6% | 16.5% | 2.4% | 0.6% | 5.2% |
| Anyksciu r. sav. | 2 | 52 | 3.9% | 0.5% | 13.2% | 2.2% | 0.5% | 4.7% |
| Druskininku sav. | 1 | 22 | 4.6% | 0.1% | 22.8% | 1.8% | 0.2% | 4.5% |
| Kupiskio r. sav. | 1 | 51 | 2.0% | 0.1% | 10.5% | 1.3% | 0.2% | 3.4% |
| Birzu r. sav. | 1 | 61 | 1.6% | 0.0% | 8.8% | 1.2% | 0.2% | 3.1% |
| Sirvintu r. sav. | 1 | 63 | 1.6% | 0.0% | 8.5% | 1.2% | 0.1% | 3.1% |
| Akmenes r. sav. | 2 | 153 | 1.3% | 0.2% | 4.6% | 1.2% | 0.3% | 2.5% |
| Siauliu r. sav. | 4 | 344 | 1.2% | 0.3% | 3.0% | 1.1% | 0.4% | 2.1% |
| Utenos r. sav. | 2 | 254 | 0.8% | 0.1% | 2.8% | 0.8% | 0.2% | 1.7% |
| Zarasu r. sav. | 1 | 128 | 0.8% | 0.0% | 4.3% | 0.8% | 0.1% | 2.0% |
| Svencioniu r. sav. | 0 | 1 | 0.0% | 0.0% | 97.5% | 0.8% | 0.0% | 3.0% |
| Varenos r. sav. | 1 | 133 | 0.8% | 0.0% | 4.1% | 0.8% | 0.1% | 2.0% |
| Alytaus r. sav. | 1 | 175 | 0.6% | 0.0% | 3.1% | 0.6% | 0.1% | 1.6% |
| Elektrenu sav. | 1 | 206 | 0.5% | 0.0% | 2.7% | 0.6% | 0.1% | 1.5% |
| Jonavos r. sav. | 0 | 40 | 0.0% | 0.0% | 8.8% | 0.5% | 0.0% | 1.9% |
| Rokiskio r. sav. | 0 | 42 | 0.0% | 0.0% | 8.4% | 0.5% | 0.0% | 1.9% |
| Pasvalio r. sav. | 0 | 44 | 0.0% | 0.0% | 8.0% | 0.5% | 0.0% | 1.8% |
| Jurbarko r. sav. | 1 | 302 | 0.3% | 0.0% | 1.8% | 0.4% | 0.1% | 1.1% |
| Moletu r. sav. | 1 | 336 | 0.3% | 0.0% | 1.7% | 0.4% | 0.1% | 1.0% |
| Salcininku r. sav. | 0 | 85 | 0.0% | 0.0% | 4.3% | 0.4% | 0.0% | 1.3% |
| Kaisiadoriu r. sav. | 1 | 409 | 0.2% | 0.0% | 1.4% | 0.3% | 0.0% | 0.8% |
| Traku r. sav. | 1 | 412 | 0.2% | 0.0% | 1.3% | 0.3% | 0.0% | 0.8% |
| Kedainiu r. sav. | 1 | 441 | 0.2% | 0.0% | 1.3% | 0.3% | 0.0% | 0.8% |
| Mazeikiu r. sav. | 1 | 460 | 0.2% | 0.0% | 1.2% | 0.3% | 0.0% | 0.8% |
| Ignalinos r. sav. | 0 | 120 | 0.0% | 0.0% | 3.0% | 0.3% | 0.0% | 1.1% |
| Radviliskio r. sav. | 1 | 503 | 0.2% | 0.0% | 1.1% | 0.3% | 0.0% | 0.7% |
| Silutes r. sav. | 0 | 135 | 0.0% | 0.0% | 2.7% | 0.3% | 0.0% | 1.0% |
| Joniskio r. sav. | 0 | 146 | 0.0% | 0.0% | 2.5% | 0.3% | 0.0% | 0.9% |
| Lazdiju r. sav. | 0 | 166 | 0.0% | 0.0% | 2.2% | 0.2% | 0.0% | 0.9% |
| Telsiu r. sav. | 1 | 799 | 0.1% | 0.0% | 0.7% | 0.2% | 0.0% | 0.5% |
| Raseiniu r. sav. | 0 | 431 | 0.0% | 0.0% | 0.9% | 0.1% | 0.0% | 0.4% |
| Kelmes r. sav. | 0 | 472 | 0.0% | 0.0% | 0.8% | 0.1% | 0.0% | 0.4% |
| Vilniaus r. sav. | 0 | 450 | 0.0% | 0.0% | 0.8% | 0.1% | 0.0% | 0.4% |
| Prienu r. sav. | 0 | 572 | 0.0% | 0.0% | 0.6% | 0.1% | 0.0% | 0.3% |
| Kauno r. sav. | 0 | 730 | 0.0% | 0.0% | 0.5% | 0.1% | 0.0% | 0.3% |

**Table S7:** Number of wild boar samples, which were investigated by ELISA to detected ASF specific antibodies and by PCR to detect ASF virus genome. Number of samples from active surveillance, which resulted in a positive PCR but a negative ELISA test result and the estimated raw and corrected prevalence (calculated using a non-spatial beta-binomial model) including the 95% confidence intervals for each ASF-affected municipality of Lithuania.

| Area | Number of wild boar showing a PCR-positive and a seronegative ASF sample | Number of samples | Raw prevalence | Lower 95% Confidence interval | Upper 95% Confidence interval | Corrected prevalence | Lower 95% Confidence interval | Upper 95% Confidence interval |
| --- | --- | --- | --- | --- | --- | --- | --- | --- |
| Pakruojo r. sav. | 10 | 131 | 7.6% | 3.7% | 13.6% | 5.3% | 3.1% | 8.0% |
| Elektrenu sav. | 10 | 206 | 4.9% | 2.4% | 8.8% | 4.0% | 2.3% | 6.0% |
| Panevezio r. sav. | 3 | 36 | 8.3% | 1.8% | 22.5% | 3.8% | 1.5% | 7.0% |
| Siauliu r. sav. | 13 | 344 | 3.8% | 2.0% | 6.4% | 3.4% | 2.1% | 5.0% |
| Kedainiu r. sav. | 16 | 441 | 3.6% | 2.1% | 5.8% | 3.4% | 2.2% | 4.7% |
| Mazeikiu r. sav. | 16 | 460 | 3.5% | 2.0% | 5.6% | 3.2% | 2.1% | 4.6% |
| Birzu r. sav. | 3 | 61 | 4.9% | 1.0% | 13.7% | 3.2% | 1.3% | 5.8% |
| Zarasu r. sav. | 5 | 128 | 3.9% | 1.3% | 8.9% | 3.1% | 1.5% | 5.3% |
| Lazdiju r. sav. | 6 | 166 | 3.6% | 1.3% | 7.7% | 3.1% | 1.5% | 5.0% |
| Alytaus r. sav. | 6 | 175 | 3.4% | 1.3% | 7.3% | 3.0% | 1.5% | 4.8% |
| Ukmerges r. sav. | 2 | 41 | 4.9% | 0.6% | 16.5% | 2.9% | 1.0% | 5.7% |
| Anyksciu r. sav. | 2 | 52 | 3.9% | 0.5% | 13.2% | 2.7% | 0.9% | 5.2% |
| Druskininku sav. | 1 | 22 | 4.6% | 0.1% | 22.8% | 2.6% | 0.7% | 5.4% |
| Akmenes r. sav. | 4 | 153 | 2.6% | 0.7% | 6.6% | 2.4% | 1.1% | 4.2% |
| Svencioniu r. sav. | 0 | 1 | 0.0% | 0.0% | 97.5% | 2.1% | 0.4% | 4.9% |
| Kupiskio r. sav. | 1 | 51 | 2.0% | 0.1% | 10.5% | 2.0% | 0.6% | 4.3% |
| Jurbarko r. sav. | 6 | 302 | 2.0% | 0.7% | 4.3% | 2.0% | 1.0% | 3.3% |
| Sirvintu r. sav. | 1 | 63 | 1.6% | 0.0% | 8.5% | 1.9% | 0.5% | 4.0% |
| Kaisiadoriu r. sav. | 7 | 409 | 1.7% | 0.7% | 3.5% | 1.8% | 0.9% | 2.9% |
| Varenos r. sav. | 2 | 133 | 1.5% | 0.2% | 5.3% | 1.7% | 0.6% | 3.4% |
| Prienu r. sav. | 8 | 572 | 1.4% | 0.6% | 2.7% | 1.5% | 0.8% | 2.3% |
| Jonavos r. sav. | 0 | 40 | 0.0% | 0.0% | 8.8% | 1.5% | 0.3% | 3.5% |
| Rokiskio r. sav. | 0 | 42 | 0.0% | 0.0% | 8.4% | 1.4% | 0.2% | 3.4% |
| Utenos r. sav. | 3 | 254 | 1.2% | 0.2% | 3.4% | 1.4% | 0.6% | 2.6% |
| Pasvalio r. sav. | 0 | 44 | 0.0% | 0.0% | 8.0% | 1.4% | 0.2% | 3.4% |
| Ignalinos r. sav. | 1 | 120 | 0.8% | 0.0% | 4.6% | 1.4% | 0.4% | 2.9% |
| Traku r. sav. | 5 | 412 | 1.2% | 0.4% | 2.8% | 1.4% | 0.6% | 2.3% |
| Radviliskio r. sav. | 6 | 503 | 1.2% | 0.4% | 2.6% | 1.3% | 0.7% | 2.2% |
| Vilniaus r. sav. | 5 | 450 | 1.1% | 0.4% | 2.6% | 1.3% | 0.6% | 2.2% |
| Joniskio r. sav. | 1 | 146 | 0.7% | 0.0% | 3.8% | 1.2% | 0.3% | 2.6% |
| Moletu r. sav. | 3 | 336 | 0.9% | 0.2% | 2.6% | 1.2% | 0.5% | 2.1% |
| Salcininku r. sav. | 0 | 85 | 0.0% | 0.0% | 4.3% | 1.1% | 0.2% | 2.6% |
| Silutes r. sav. | 0 | 135 | 0.0% | 0.0% | 2.7% | 0.9% | 0.1% | 2.0% |
| Kauno r. sav. | 4 | 730 | 0.6% | 0.2% | 1.4% | 0.7% | 0.3% | 1.3% |
| Telsiu r. sav. | 4 | 799 | 0.5% | 0.1% | 1.3% | 0.7% | 0.3% | 1.2% |
| Raseiniu r. sav. | 1 | 431 | 0.2% | 0.0% | 1.3% | 0.6% | 0.2% | 1.2% |
| Kelmes r. sav. | 1 | 472 | 0.2% | 0.0% | 1.2% | 0.5% | 0.1% | 1.1% |

**Table S8:** Number of wild boar samples, which were investigated only by PCR to detect ASF virus genome. Number of samples from passive surveillance, which resulted in a positive PCR test result and the estimated raw and corrected prevalence (calculated using a non-spatial beta-binomial model) including the 95% confidence intervals for each ASF-affected municipality of Lithuania.

| Area | Number of wild boar showing a PCR-positive ASF sample | Number of samples | Raw prevalence | Lower 95% Confidence interval | Upper 95% Confidence interval | Corrected prevalence | Lower 95% Confidence interval | Upper 95% Confidence interval |
| --- | --- | --- | --- | --- | --- | --- | --- | --- |
| Panevezio r. sav. | 357 | 359 | 99.4% | 98.0% | 99.9% | 99.1% | 98.1% | 99.7% |
| Pasvalio r. sav. | 71 | 76 | 93.4% | 85.3% | 97.8% | 92.1% | 86.6% | 96.3% |
| Lazdiju r. sav. | 189 | 209 | 90.4% | 85.6% | 94.1% | 90.0% | 86.4% | 93.1% |
| Pakruojo r. sav. | 71 | 78 | 91.0% | 82.4% | 96.3% | 89.8% | 83.9% | 94.7% |
| Zarasu r. sav. | 43 | 47 | 91.5% | 79.6% | 97.6% | 89.6% | 81.9% | 95.5% |
| Anyksciu r. sav. | 107 | 119 | 89.9% | 83.1% | 94.7% | 89.2% | 84.3% | 93.4% |
| Druskininku sav. | 8 | 8 | 100.0% | 63.1% | 100.0% | 89.0% | 72.7% | 98.6% |
| Sirvintu r. sav. | 231 | 263 | 87.8% | 83.3% | 91.5% | 87.5% | 84.1% | 90.7% |
| Ukmerges r. sav. | 282 | 325 | 86.8% | 82.6% | 90.3% | 86.5% | 83.3% | 89.5% |
| Elektrenu sav. | 84 | 100 | 84.0% | 75.3% | 90.6% | 83.4% | 77.1% | 89.0% |
| Alytaus r. sav. | 38 | 45 | 84.4% | 70.5% | 93.5% | 83.2% | 73.9% | 91.0% |
| Kauno r. sav. | 51 | 61 | 83.6% | 71.9% | 91.9% | 82.7% | 74.6% | 89.7% |
| Ignalinos r. sav. | 17 | 20 | 85.0% | 62.1% | 96.8% | 82.4% | 68.8% | 93.0% |
| Jurbarko r. sav. | 227 | 275 | 82.6% | 77.5% | 86.8% | 82.4% | 78.5% | 86.0% |
| Rokiskio r. sav. | 16 | 19 | 84.2% | 60.4% | 96.6% | 81.6% | 67.6% | 92.7% |
| Prienu r. sav. | 41 | 51 | 80.4% | 66.9% | 90.2% | 79.6% | 70.3% | 87.8% |
| Radviliskio r. sav. | 58 | 75 | 77.3% | 66.2% | 86.2% | 77.0% | 69.0% | 84.3% |
| Siauliu r. sav. | 101 | 131 | 77.1% | 69.0% | 84.0% | 76.9% | 70.8% | 82.6% |
| Kelmes r. sav. | 1 | 1 | 100.0% | 2.5% | 100.0% | 76.8% | 46.8% | 96.8% |
| Telsiu r. sav. | 34 | 45 | 75.6% | 60.5% | 87.1% | 75.2% | 64.8% | 84.5% |
| Akmenes r. sav. | 73 | 97 | 75.3% | 65.5% | 83.5% | 75.1% | 67.8% | 81.8% |
| Kedainiu r. sav. | 69 | 93 | 74.2% | 64.1% | 82.7% | 74.1% | 66.6% | 81.0% |
| Mazeikiu r. sav. | 48 | 66 | 72.7% | 60.4% | 83.0% | 72.7% | 63.7% | 81.0% |
| Traku r. sav. | 18 | 26 | 69.2% | 48.2% | 85.7% | 69.8% | 55.8% | 82.3% |
| Jonavos r. sav. | 22 | 32 | 68.8% | 50.0% | 83.9% | 69.3% | 56.4% | 80.9% |
| Utenos r. sav. | 14 | 22 | 63.6% | 40.7% | 82.8% | 65.4% | 50.0% | 79.4% |
| Birzu r. sav. | 10 | 16 | 62.5% | 35.4% | 84.8% | 65.0% | 47.6% | 80.8% |
| Moletu r. sav. | 50 | 83 | 60.2% | 48.9% | 70.8% | 61.0% | 52.3% | 69.3% |
| Varenos r. sav. | 14 | 24 | 58.3% | 36.6% | 77.9% | 60.9% | 45.9% | 75.1% |
| Kaisiadoriu r. sav. | 32 | 56 | 57.1% | 43.2% | 70.3% | 58.5% | 48.0% | 68.6% |
| Vilniaus r. sav. | 21 | 39 | 53.9% | 37.2% | 69.9% | 56.1% | 43.8% | 68.1% |
| Silutes r. sav. | 1 | 4 | 25.0% | 0.6% | 80.6% | 52.1% | 26.2% | 77.5% |
| Kupiskio r. sav. | 3 | 8 | 37.5% | 8.5% | 75.5% | 51.5% | 29.6% | 73.1% |
| Raseiniu r. sav. | 21 | 44 | 47.7% | 32.5% | 63.3% | 50.4% | 38.8% | 62.0% |
| Joniskio r. sav. | 54 | 113 | 47.8% | 38.3% | 57.4% | 48.9% | 41.4% | 56.4% |
| Salcininku r. sav. | 1 | 6 | 16.7% | 0.4% | 64.1% | 42.9% | 20.4% | 66.9% |
| Svencioniu r. sav. | 35 | 172 | 20.4% | 14.6% | 27.2% | 21.9% | 17.0% | 27.2% |

**
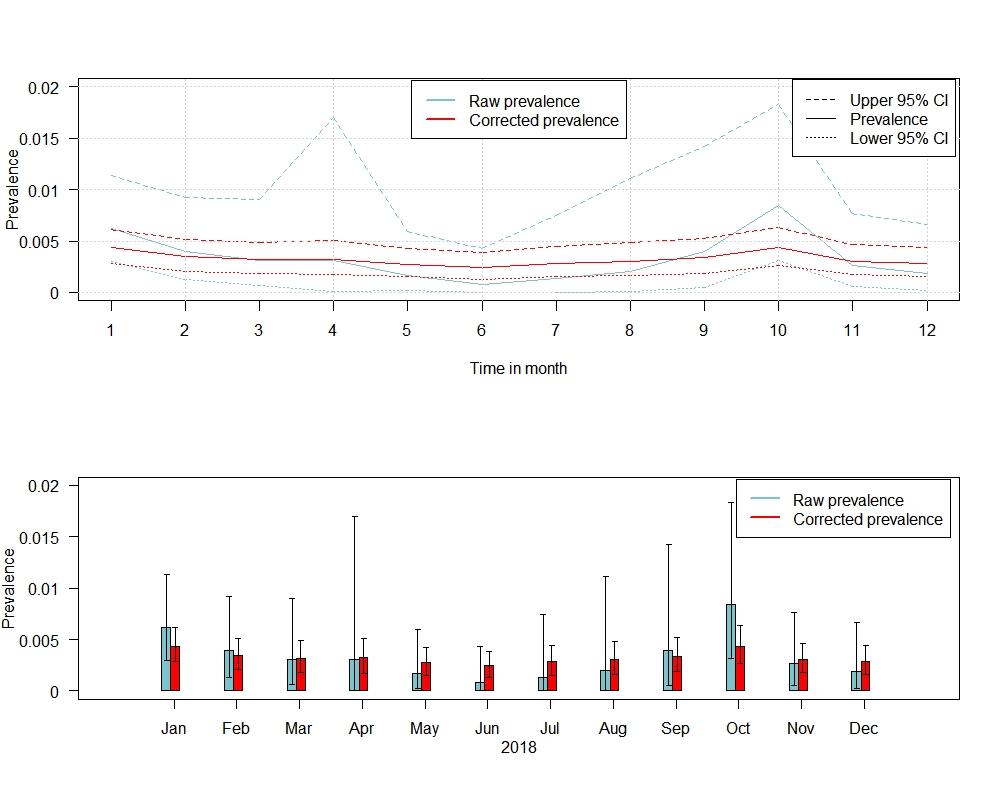
**

**Figure S1:** Estimated raw and corrected prevalence (calculated using a non-spatial beta-binomial model) of hunted wild boar showing an ELISA and a PCR-positive test result for each month of 2018. The whiskers indicate 95% confidence intervals.


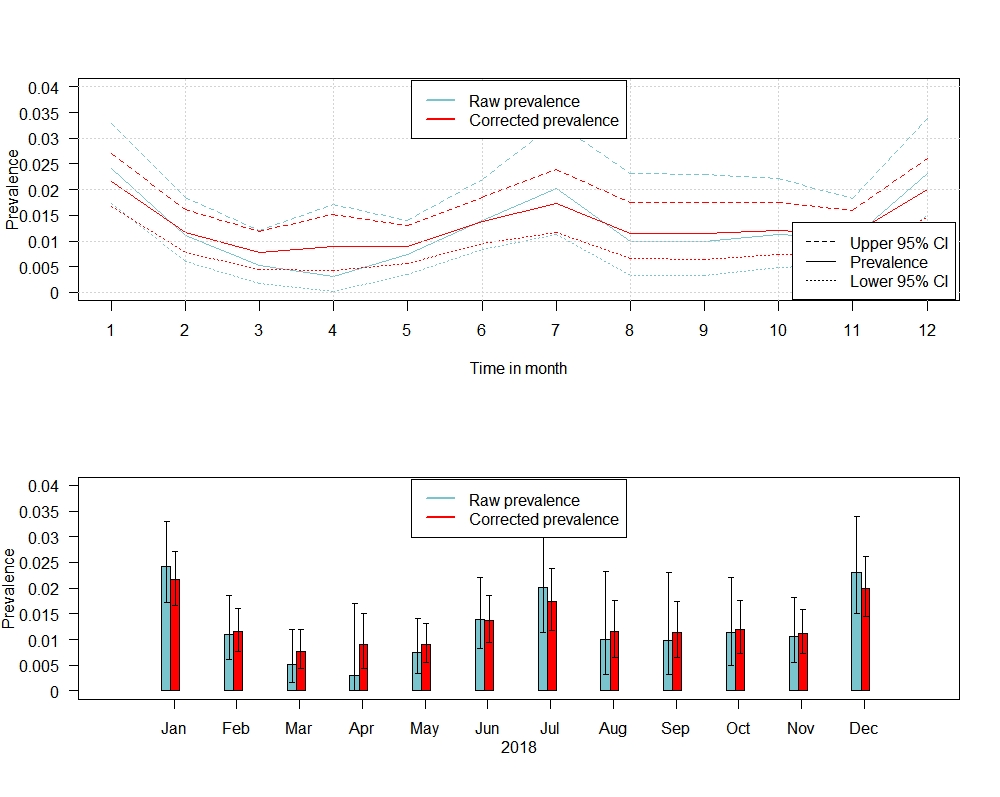


**Figure S2:** Estimated raw and corrected prevalence (calculated using a non-spatial beta-binomial model) of hunted wild boar showing an ELISA-negative but a PCR-positive test result for each month of 2018. The whiskers indicate 95% confidence intervals.


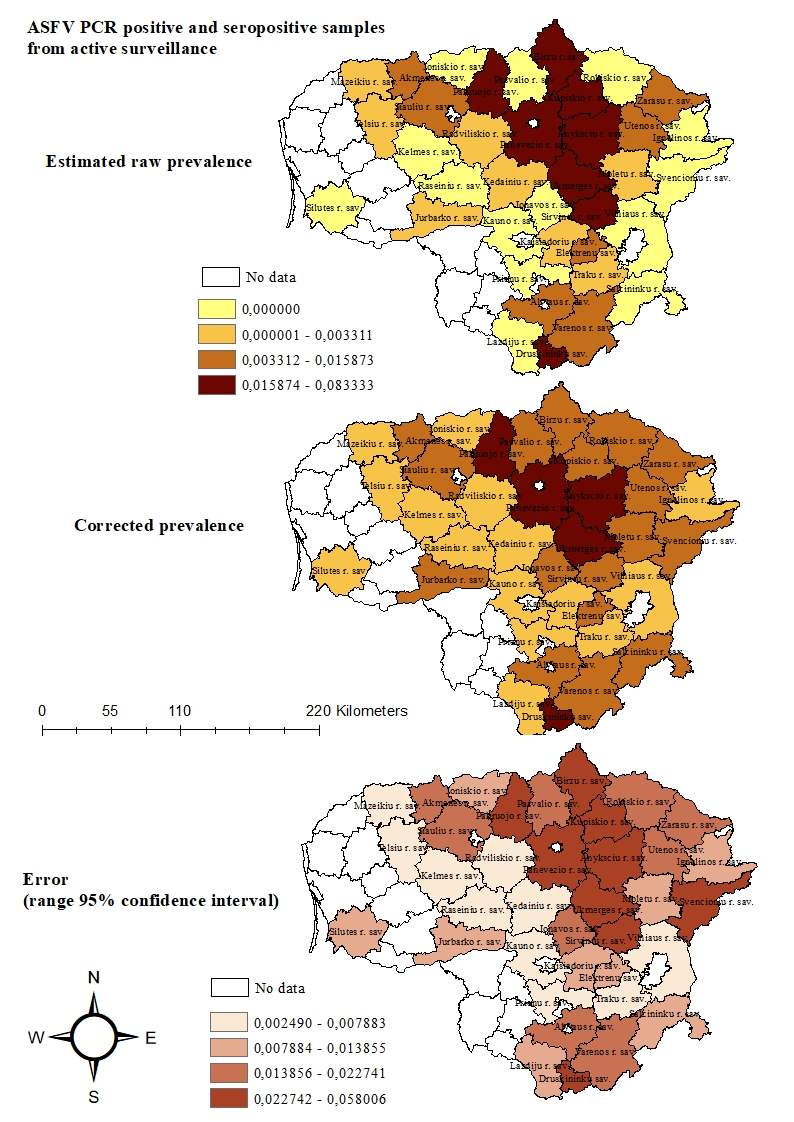


**Figure S3:** Estimated raw and corrected prevalence with 95% confidence intervals (calculated using a non-spatial beta-binomial model) of hunted wild boar showing a sero- and PCR- positive ASF sample result for each ASF-affected municipality of Lithuania.


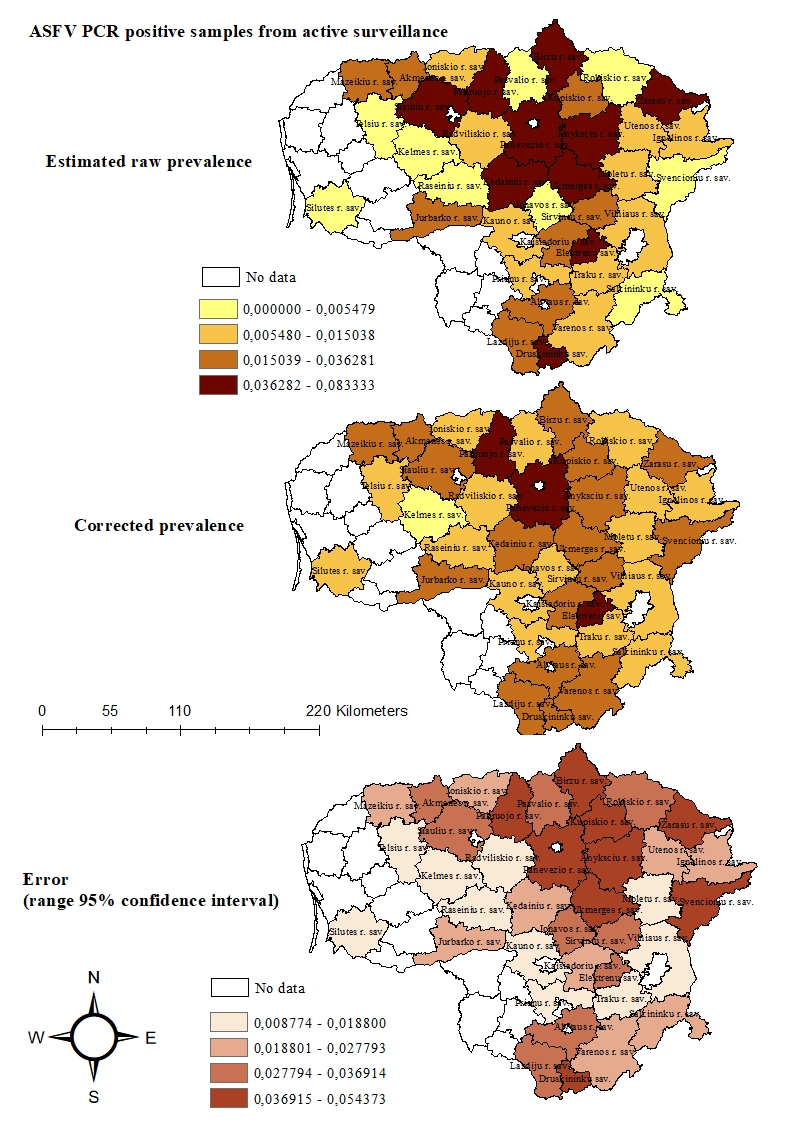


**Figure S4:** Estimated raw and corrected prevalence with 95% confidence intervals (calculated using a non-spatial beta-binomial model) of hunted wild boar showing a PCR- positive and a seronegative ASF sample result for each ASF-affected municipality of Lithuania.


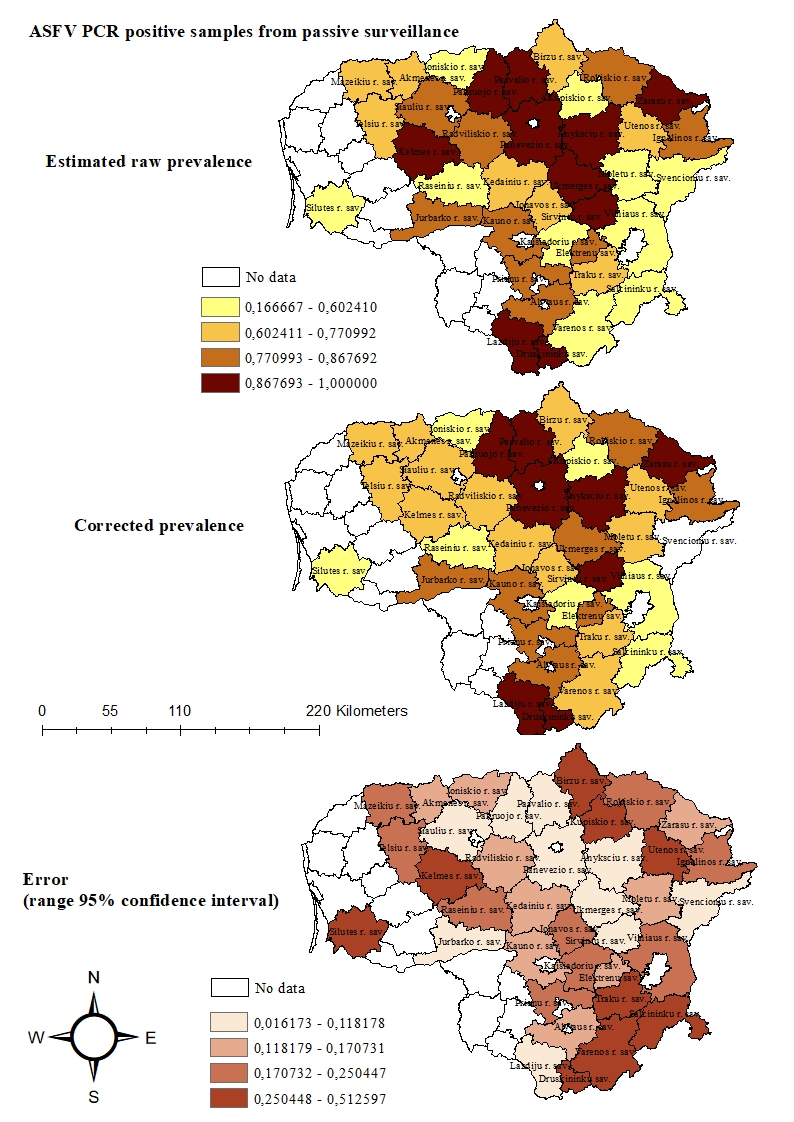


**Figure S5:** Estimated raw and corrected prevalence with 95% confidence intervals (calculated using a non-spatial beta-binomial model) of wild boar found dead showing a PCR- positive ASF sample result for each ASF-affected municipality of Lithuania.
